# Serum ganciclovir drug exposure in children receiving standard ganciclovir dosing

**DOI:** 10.1128/aac.00525-24

**Published:** 2024-09-18

**Authors:** Wenyu Yang, Adam Irwin, Heather Weerdenburg, Brett McWhinney, Theresa Cole, Alice Lei, Bing Han, Xiao Zhu, Amanda Gwee

**Affiliations:** 1 Minhang Hospital & School of Pharmacy, Fudan University, Shanghai, China; 2 Centre for Clinical Research, The University of Queensland, Brisbane, Queensland, Australia; 3 Infection Management and Prevention Service, Queensland Children’s Hospital, Brisbane, Queensland, Australia; 4 Children’s Cancer Centre, Royal Children’s Hospital, Melbourne, Victoria, Australia; 5 Department of Paediatrics, The University of Melbourne, Melbourne, Victoria, Australia; 6 Antimicrobial Group, Murdoch Children’s Research Institute, Victoria, Australia; 7 Department of Chemical Pathology, Pathology Queensland, Royal Brisbane and Women’s Hospital, Brisbane, Queensland, Australia; 8 Department of Haematology, Royal Children’s Hospital, Melbourne, Melbourne, Victoria, Australia; 9 Department of General Medicine, Royal Children’s Hospital, Melbourne, Victoria, Australia; 10 Department of Pharmacy, Minhang Hospital, Fudan University, Shanghai, China; University Children's Hospital Münster, Münster, Germany

**Keywords:** external validation, ganciclovir, model-informed precision dosing, pediatrics, population pharmacokinetics

## Abstract

Intravenous ganciclovir (GCV) is used for the treatment of cytomegalovirus (CMV) infection in immunocompromised children. Although the therapeutic target for treatment is unclear, studies have shown a serum area under the concentration–time curve (AUC_24h_) ≥40 mg/L·h correlates with effective CMV prevention. This study aimed to externally validate existing GCV population pharmacokinetic (PopPK) models and develop a model if needed and evaluate the serum AUC_24h_ achieved with standard GCV dosing and propose an optimized dosing strategy for immunocompromised children. Ganciclovir drug monitoring data from two pediatric hospitals were retrospectively collected, and published pediatric PopPK models were externally validated. The population AUC_24h_ with standard GCV dosing (5 mg/kg twice daily) was calculated, and an optimized dosing strategy was determined using Monte Carlo simulations to achieve an AUC_24h_ between 40 and 100 mg/L·h. Overall, 161 samples from 23 children with a median (range) age of 9.0 years (0.4–17.0) and weight of 28.2 kg (5.6–73.3) were analyzed. Transferability of published pediatric PopPK models was limited. Thus, a one-compartment model with first-order absorption and elimination with weight and serum creatinine as covariates was developed. The median (5th–95th percentiles) steady state AUC_24h_ with standard dosing was 38.3 mg/L·h (24.8–329.2) with 13 children having an AUC_24h_ <40 mg/L·h, particularly those aged <4 years (8/13). An optimized simulated GCV dosing regimen, ranging from 2 to 13 mg/kg twice daily for children with normal renal function, achieved 61%–78% probability of target attainment. Standard GCV dosing likely results in inadequate drug exposure in more than half of the children, particularly those aged <4 years. An optimized dosing regimen has been proposed for clinical validation.

## INTRODUCTION

Cytomegalovirus (CMV) remains a significant threat to the well-being and survival of immunocompromised children, in particular allogeneic hematopoietic stem cell transplant (HSCT) recipients (1). Due to the risk of severe end-organ disease (pneumonitis, hepatitis, colitis, nephritis, and/or retinitis), CMV-related mortality rates of up to 40% have been reported (2, 3). The established primary treatment for CMV infection in children is intravenous ganciclovir (IV GCV) at a dose of 5 mg/kg twice daily (4–7). However, recent studies have reported wide variability in serum drug exposure with this dose among children, highlighting the potential role of therapeutic drug monitoring and dose optimization (8).

Although the therapeutic targets for GCV/VGCV treatment of CMV infection and disease have not been well defined, breakthrough CMV infection in patients receiving GCV prophylaxis has been reported when serum drug exposure, as measured by the area under the concentration–time curve over a 24-h period (AUC_24h_), is below 40 mg/L·h (9, 10). It is possible that low serum GCV exposures may contribute to the longer duration of CMV viremia observed in immunocompromised children in comparison to adults (11, 12). Unfortunately, there are few population pharmacokinetic (PopPK) studies in children and only one reporting the drug exposure achieved with standard GCV dosing (13).

Therefore, this study aims to assess the transferability of published PopPK models for our study data set derived from immunocompromised children; if required, develop a PopPK model of GCV/VGCV; determine the GCV drug exposure achieved with the standard GCV dosing regimen; and provide an optimized dosing regimen to improve target attainment in children with normal renal function.

## MATERIALS AND METHODS

### Clinical audit

This study was a multicenter retrospective audit of immunocompromised children receiving GCV or VGCV for prophylaxis or treatment of CMV infection at The Royal Children’s Hospital Melbourne or Queensland Children’s Hospital Brisbane, Australia, over a 58-month period (April 2018 to January 2023). Both centers are tertiary pediatric hospitals and the primary referral center for pediatric HSCT for their state. Eligible children were identified with an electronic medical record report and included if they were immunocompromised, aged 0 to 18 years, received GCV or VGCV, and had serum GCV concentrations determined. Immunocompromised children included those with malignancy (hematological, solid organ), hematopoietic stem cell transplant or solid organ transplant recipients, primary immunodeficiency, or those receiving systemic immunosuppression (e.g., inflammatory bowel disease). Neonates with congenital CMV were excluded.

Demographic and clinical data were collected from the medical record and included age, sex, underlying diagnosis, body weight, height, serum creatinine (SCR), albumin (ALB), white cell count (WCC), as well as start and end dates of renal replacement therapy. Body surface area (BSA) was calculated using the Mosteller formula (14). At these hospitals, the standard GCV dosing regimen is 5 mg/kg twice a day (bid) administered as a 1-h infusion. The VGCV dose is 16 mg/kg bid for neonates, and 7 × BSA × creatinine clearance (CrCL) bid outside the neonatal period (administered with food when possible). At Queensland Children’s Hospital, a dose of 16 mg/kg bid is used in infants aged 1 month to 1 year. At both centers, two serum GCV concentrations were routinely determined (0.5 mL of blood per sample): a trough concentration taken 30 min to 1 h before the dose; and peak at 1 to 4 h post-GCV. Blood samples were centrifuged at 5300 rpm for 5 min, after which samples were stored at 4°C until analysis, which was conducted either on the same day or the following day. Serum GCV concentrations were quantified at Queensland Pathology using a validated ultra performance liquid chromatography (UPLC) coupled with QDa mass detection (Waters Corporation, Milford, MA, USA). The lower limit of quantification (LLOQ) was 0.2 mg/L, linearity was up to 25 mg/L, and the inter-run imprecision was <7% across two quality control levels (0.7 and 7.0 mg/L).

### External validation of published pediatric GCV/VGCV pharmacokinetic models

Pediatric GCV/VGCV models identified (Fig. S1) from our previous literature review were externally validated using the study data set (15). MAXEVAL = 0 option in nonlinear mixed-effects modeling (NONMEM) software (version 7.5, ICON Development Solutions, Ellicott City, MD) was used for external evaluation. Outputs from NONMEM were assessed using Perl-speaks-NONMEM (PsN, version 5.2.6, Uppsala University, Sweden). Pirana (version 23.1.2, Certara, United States) was utilized to manage the modeling workflow, and R (version 4.2.2, http://www.r-project.org) was utilized for statistical and graphical diagnostics (Supplementary materials). The model’s population predictive performance was deemed acceptable if it achieved a root mean square error (RMSE) capped at 2 mg/L (16, 17).

### Population pharmacokinetic model development

PopPK parameters were estimated using first-order conditional estimation with interaction (FOCE-I) method in NONMEM. If the percentage of below quantification limit (BQL) data was <10%, BQL data were excluded during the modeling process (18). One- and two-compartment models with first-order absorption were investigated. Inter-individual variability (IIV) was assessed using exponential error models. Additive, proportional and combined residual error models were examined.

Given that GCV is a renally cleared, renal function was incorporated as a significant covariate of GCV clearance (CL). SCR was tested *a priori* as a time-varying covariate through a power function. Biologically plausible covariates were assessed through stepwise covariate modeling. During the forward selection, a reduction in the objective function value (OFV) of at least 3.84 (*P* < 0.05) was considered significant. For backward elimination, an increase in OFV of ≥10.83 (*P* < 0.001) was necessary for retention in the final model. Allometric scaling to a 70-kg individual (fixed allometric exponents) was also tested and compared (19, 20).

To identify clinically relevant covariates on steady state AUC_24h_ (AUC_ss,24h_), the impact of marginal effects changing one covariate at a time on AUC was explored using the R package "coveffectsplot" (21).

### Drug exposure calculation

The individual AUC_ss,24h_ of included children was determined by the individual’s CL and standard GCV dosing. We also simulated the drug exposure achieved with standard dosing drug exposure across four age groups from a covariate database of a larger cohort of 703 children aged 1–18 years old from The Royal Children’s Hospital Melbourne.

### Model-based dose optimization

The final PopPK model was used to perform Monte Carlo simulations for a series of weight-based dosage regimens of GCV. An AUC_ss,24h_ of 40–100 mg/L·h was adopted as the surrogate pharmacokinetic/pharmacodynamic (PK/PD) target (the rationale for this range presented in the Discussion). Given that children’s renal function closely correlates with age, each age group was subdivided into normal and elevated SCR subgroups (*n* = 1000). The normal SCR distribution was derived from a covariate database of 703 children (aged 1–18 years) at The Royal Children’s Hospital Melbourne. To determine their weights, the observed correlation between weight and age within our patient cohort was used. Dose regimens within 5% of the highest probability of target attainment (PTA) were considered for recommendation. Then, the dose that simultaneously achieved high PTAs across various age and SCR subgroups was recommended. The PTA of the standard and optimized dose regimen for children with normal renal function was then compared using the aforementioned covariate database (resample, *n* = 1000).

## RESULTS

### Data characteristics

During the study period, 24 immunocompromised children had 176 serum GCV concentrations with a median of two samples (range, 1–53) per child. These children had a median age of 9.0 years (range, 0.42–17.0), median weight of 27.4 kg (range, 5.6–73.3) and median baseline SCR level of 30 µmol/L (range, 19–167 μmol/L) (Table 1). Notably, two (8%) were infants aged <1 years. Fifteen (9%) of the GCV concentrations were below the LLOQ and excluded from data analysis leaving 161 serum concentrations from 23 children for analysis.

**TABLE 1:** Characteristics of the population at baseline^*a*^

Characteristics	Number of patients (%) Median (min–max)
Sex (female/male)	11/12
RRT (yes/no)	4/19
Administration route	
IV GCV	13 (56.5%)
Enteral VGCV	1 (4.3%)
IV GCV + enteral VGCV	9 (39.1%)
Age (years)	9.0 (0.4–17.0)
0–<2	2 (8.7%)
2–< 6	7 (30.4%)
6–< 12	9 (39.1%)
12–< 18	5 (21.7%)
GCV concentration	*n* = 161 1.9 mg/L (0.2–18.7)
Samples after GCV administration	*n* = 113 (70.2%) 1.7 mg/L (0.2–14.2)
Samples after VGCV administration	*n* = 48 (29.8%) 2.3 mg/L (0.2–18.7)
GCV dose (mg/kg/dose)	5 (0.6–10.9)
VGCV dose (mg/kg/dose)	17.8 (6.0–47.4)
Weight (kg)	28.2 (5.6–73.3)
Height (cm)	132.0 (60.5–170.0)
Body surface area (m^2^)	1.01 (0.31–1.84)
Serum creatinine (μmol/L)	30.0 (19.0–167)
KF	0.82 (0.16–1.07)
CrCL (mL/min/1.73 m^2^)	145 (37.2–491)

^*a*^ CrCL: creatinine clearance, calculated by modified Schwartz formula; RRT: renal replacement therapy. GCV: ganciclovir; IV: intravenous infusion administration; KF: kidney function, a dimensionless parameter, calculated as dividing CrCL by the normal renal function (120 mL/min/1.73 m^2^), where CrCL was calculated using the Gao formula (22); VGCV: valganciclovir.

### Transferability of published pediatric models

Five published models were externally validated (Table S2) (8, 23–26). Two models were of IV GCV only (23, 25), whereas the remaining three models (8, 24, 26) included patients receiving both GCV and VGCV. All five models exhibited RMSE exceeding 2 mg/L (Table 2), and diagnosis plots indicated that these models failed to adequately describe our data (Fig. S2 and S3).

**TABLE 2:** Predictive performance of published population pharmacokinetic models of ganciclovir based on population predictions^*a*^

Model	MPE (mg/L)	MAPE (mg/L)	RMSE (mg/L)
Zhou et al. (23)	10.81	10.81	14.32
Acosta et al. (24)	−2.42	2.49	3.58
Li et al. (25)	−1.70	1.87	2.59
Franck et al. (8)	−1.29	1.71	2.32
Nguyen et al. (26)	−0.63	1.48	2.10

^*a*^ MAPE: mean absolute prediction error; MPE: mean prediction error; RMSE: root mean square error.

### Final population pharmacokinetic model

**Fig 1:**
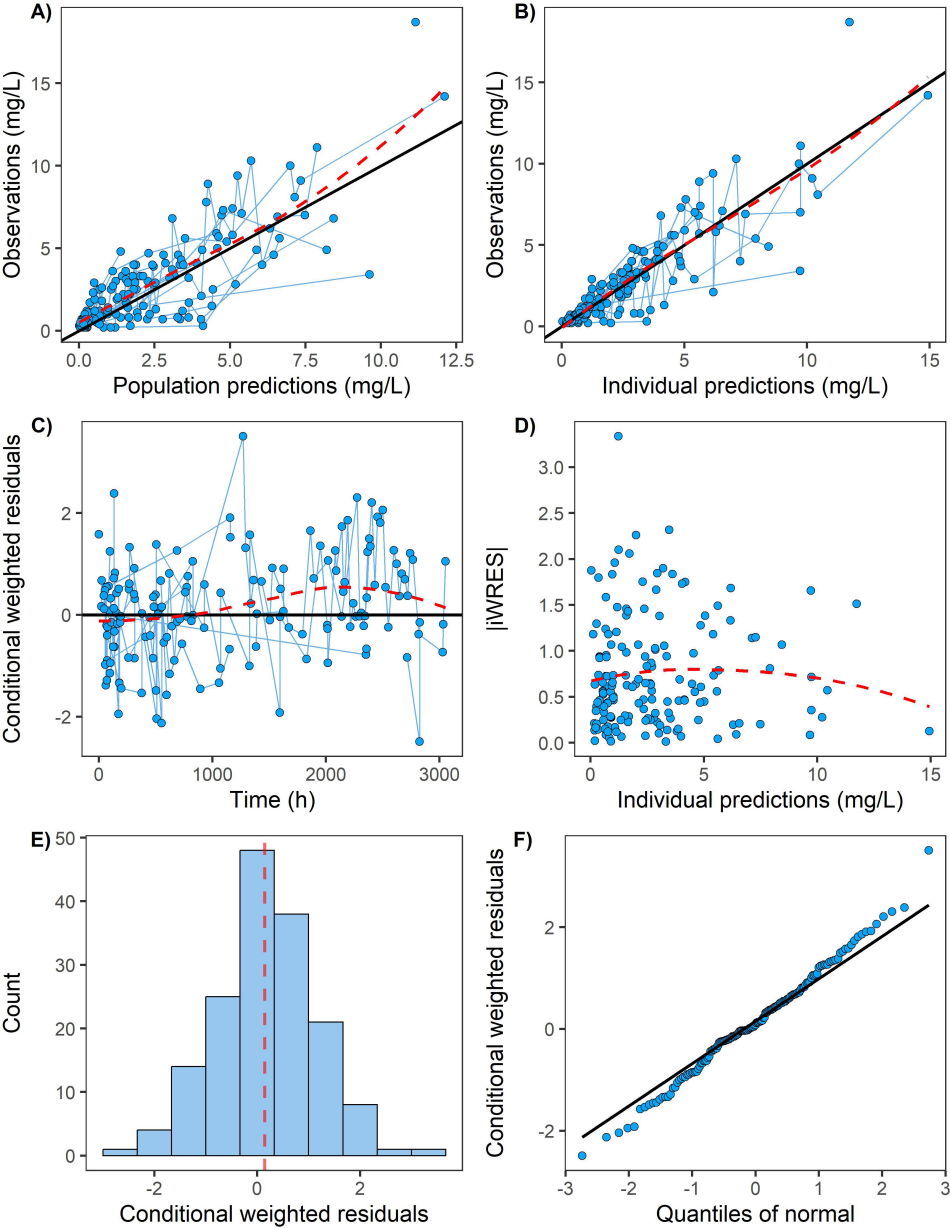
Goodness-of-fit plot of the final model. iWRES: individual conditional weighted residuals. (A) and (B) show observations vs population and individual predictions of ganciclovir concentration, respectively. The black solid line is the reference line for Y = X. The red dotted line is the loess curve of these data; the trend line and the reference line have a high degree of coincidence, and these points are also concentrated around the reference line, which indicates that the population predictions and individual predictions of our model are close to the observations; in panels (C) and (D), the reference line is the black solid line of Y = 0. The red trend line is relatively stable, and the data points are symmetrically distributed on both sides of the reference line; panels (E) and (F) prove the normal distribution of our data.

**Fig 2:**
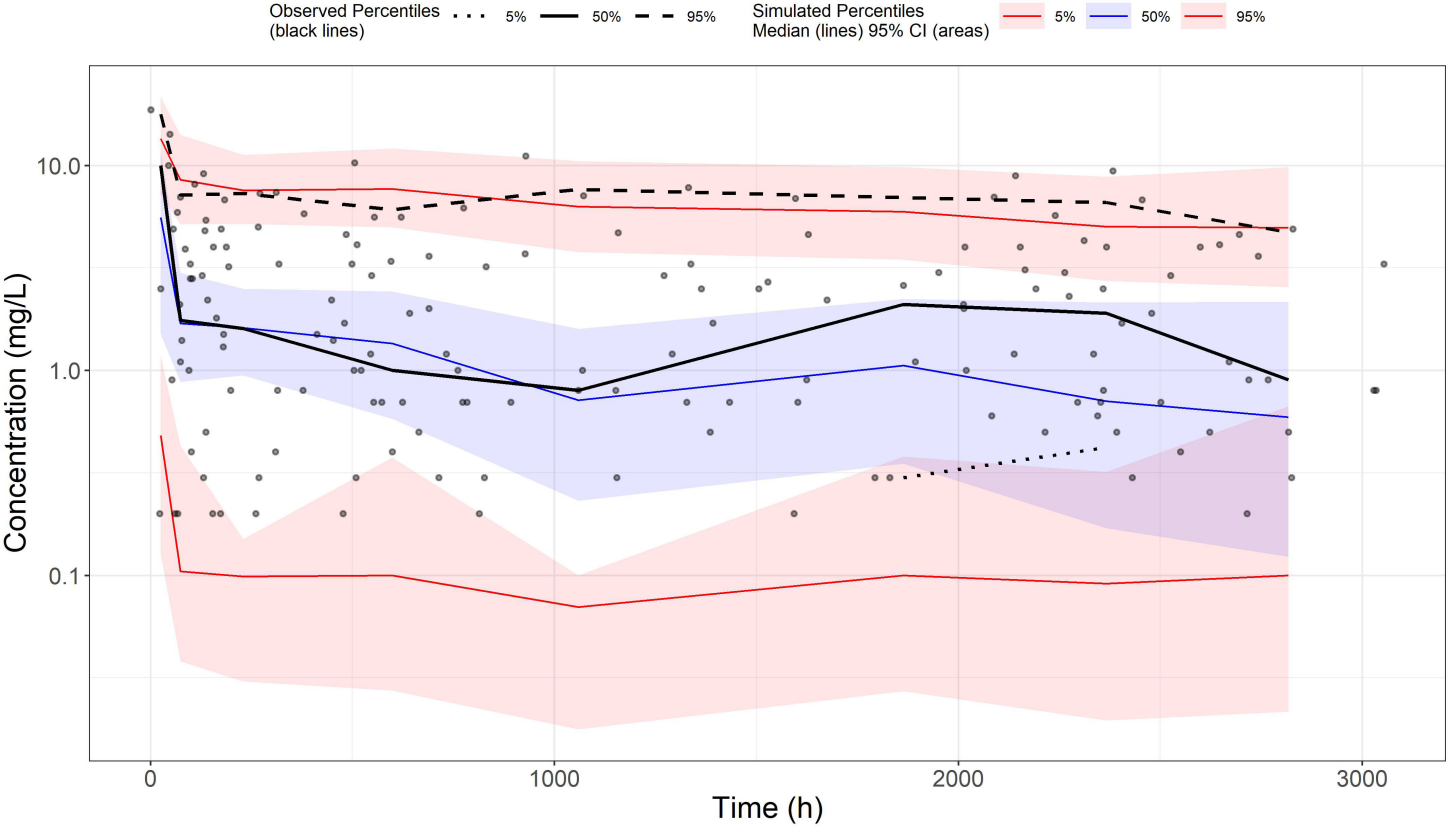
Visual predictive check of the final model. The black line represents the median observed data, and the two black dashed lines represent the 5th and 95th percentages of observed data, respectively. The light blue and light red areas represent the 95% CI for the median and the 5th and 95th percentiles of the simulated data, respectively.

Bioavailability (F) could not be accurately estimated with a relative standard error (RSE) of 46% (Table S3) and was fixed at 0.536 (after dividing by the conversion ratio of GCV and VGCV of 0.72, the absolute bioavailability was 0.744) according to a previous study (24). The final model was a one-compartment model with first-order absorption and first-order elimination (Fig. 1 and 2). Each parameter was estimated precisely with RSE lower than 30%, and all parameter estimates were close to their medians of bootstrap analysis (Table 3), indicating that the final model was robust. Significant covariates included weight and SCR for CL, as well as weight for the volume of distribution of the central compartment (V). CL and V were scaled to a 70kg individual with fixed exponents of 0.75 and 1, respectively.

**TABLE 3:** Final population pharmacokinetic model parameter estimates of ganciclovir and bootstrap results^*a*^

Fixed effect parameters	Final model	Bootstrap (*n* = 1000)
	Estimates (RSE) (Shrinkage)	Median (2.5th–97.5th)
CL (L/h)	14.1 (9%)	14.0 (11.9–16.6)
The effect of SCR on CL	0.997 (7%)	0.995 (0.687–1.12)
The effect of weight on CL	0.75 FIX	/
V (L)	62.5 (4%)	62.7 (55.3–69.1)
The effect of weight on V	1 FIX	/
Ka (h^−1^)	0.66 (25%)	0.670 (0.186–1.55)
^*b*^F	0.536 FIX	/
Between-subject variability		
CL (CV%)	36.6% (15%) (10%)	34.7% (17.0%–45.9%)
Residual unexplained variability		
prop.error.sd	39.2% (7%) (5%)	39.2% (30.2%–43.7%)
add.error.sd (mg/L)	0.144 (21%) (5%)	0.138 (0.048–0.199)

^*a*^ CL: clearance; CV%: coefficient of variability, expressed as $\sqrt{omega^{2}}\times 100\%$; F: bioavailability; Ka: absorption rate constant; RSE:relative standard error; SCR: serum creatinine (μmol/L); V: central volume of distribution.$CL=14.10\times {\left(\frac{30}{SCR}\right)}^{0.997}\times {\left(\frac{weight}{70}\right)}^{0.75}.\;V=62.5\times \frac{weight}{70}.$

^*b*^ As the dose administered was recorded as milligrams of VGCV, the value of fixed F included the ratio between the molecular weights of GCV and VGCV (which equals to 0.72), the absolute bioavailability of VGCV, and the potentially incomplete conversion of VGCV to GCV. After adjustment by dividing the ratio, the absolute bioavailability after enteral VGCV was 0.744.

**Fig 3:**
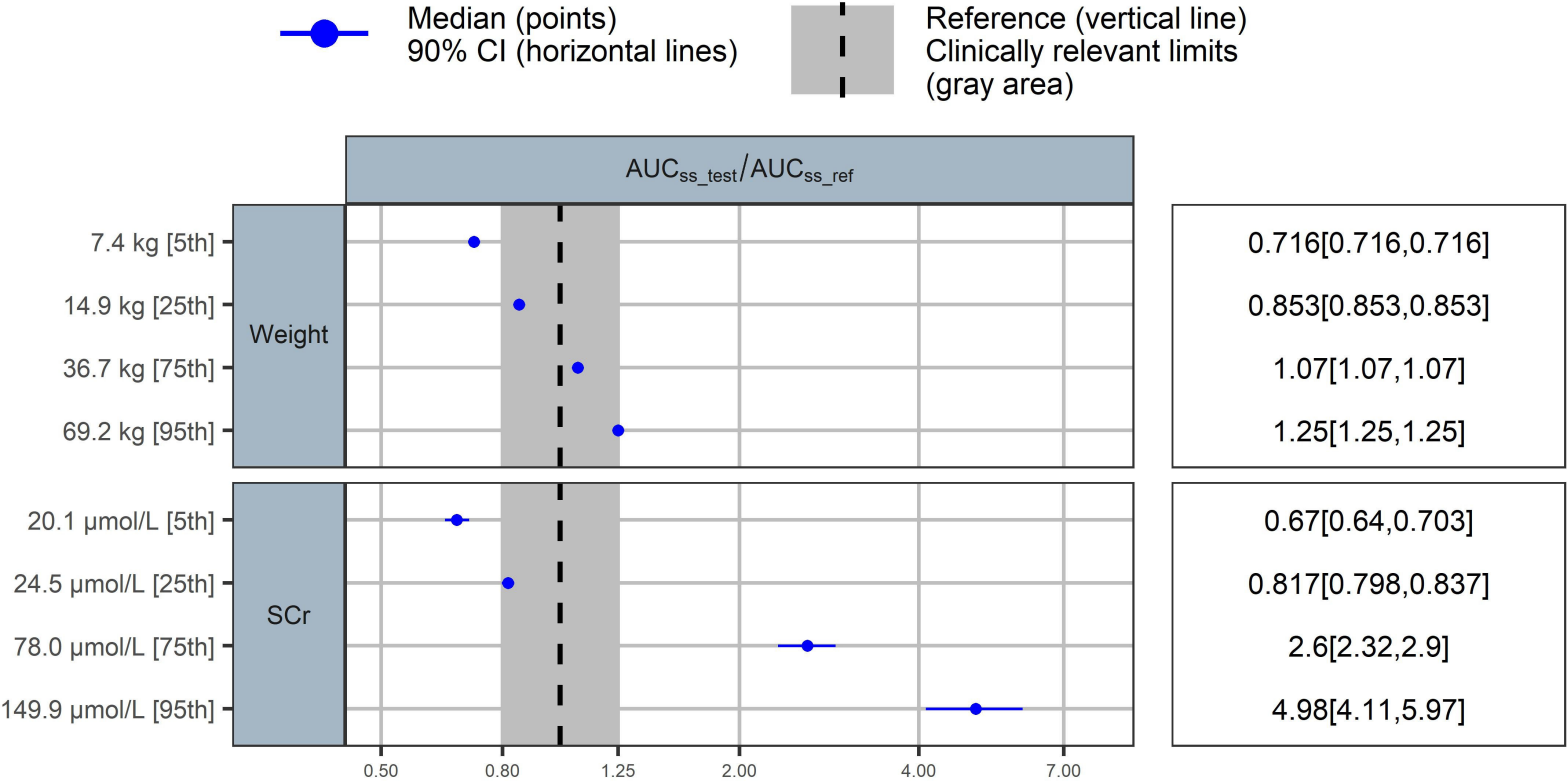
Covariate effects on steady state AUC_24h_ under the standard ganciclovir dosing (5 mg/kg twice daily). The blue circles represent the point estimates of covariate effect relative to the reference (typical patient: weight 28.2 kg, SCR 30 µmol/L), and the associated horizontal lines are the 90% confidence intervals. The shaded area ranges from 0.8 to 1.25. Within this area, the covariate effect is not clinically relevant.

A weight-based dosing strategy could account for the minimal variability seen in AUC_ss,24h_ within the 5th to 95th centile of weight values observed in the included children (i.e., from 14.9 to 69.2 kg, the change in AUC_ss,24h_ was not significant) (Fig. 3). By contrast, a fivefold increase in SCR corresponded to a fivefold rise in AUC_ss,24h_, highlighting the need to incorporate renal function into the optimized dosing regimen.

### Drug exposure

**Fig 4:**
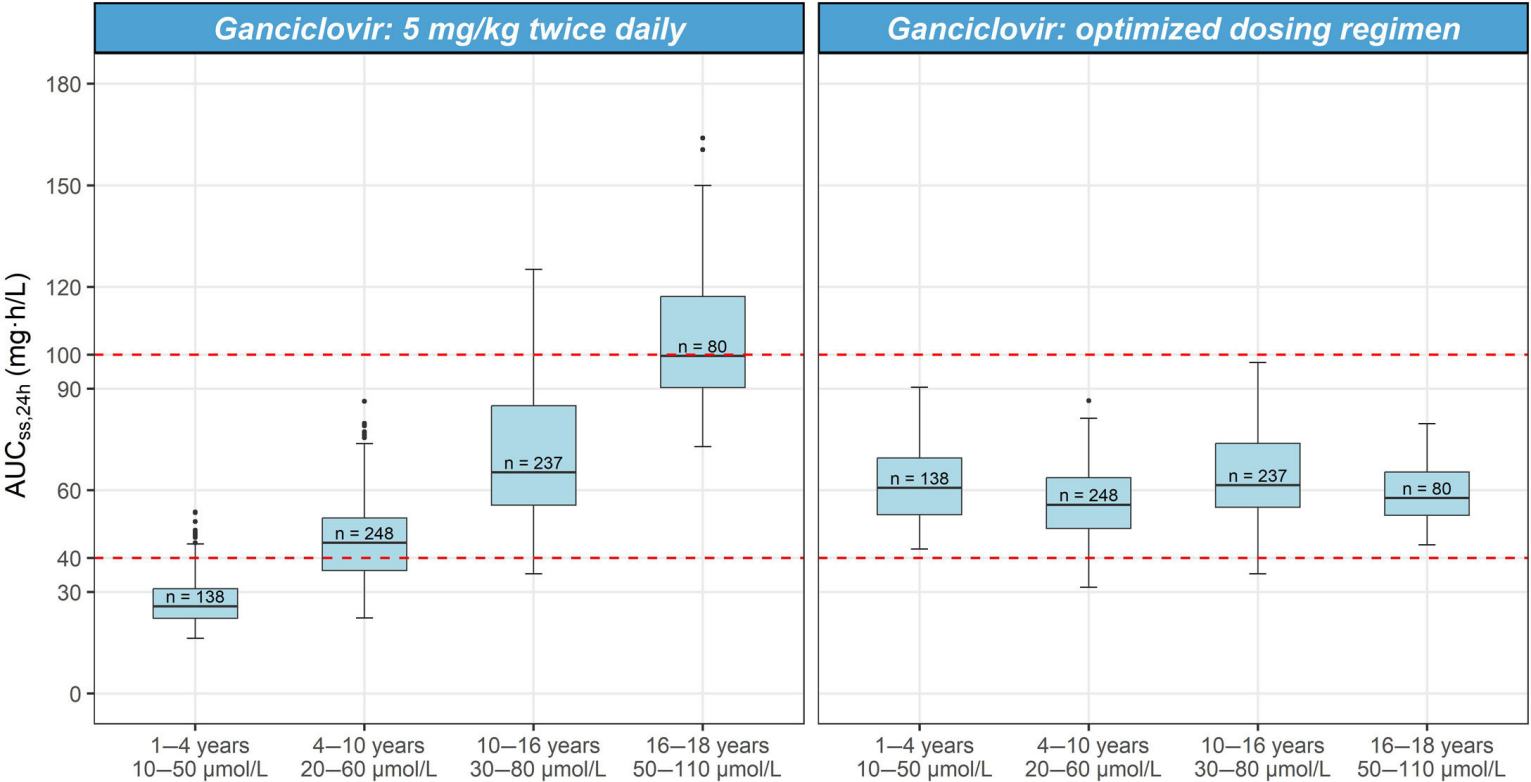
Simulated ganciclovir exposure of standard ganciclovir dosing (5 mg/kg twice daily) and optimized dosing regimen for different age groups with normal serum creatinine. Data of the 703 children from the covariate database of The Royal Children’s Hospital Melbourne. The age-related normal serum creatinine distribution was derived from the covariate database. The red dashed lines represent the target AUC_ss,24h_ range from 40 to 100 mg/L·h.

The median (5th–95th percentiles) *post hoc* individual GCV AUC_ss,24h_ estimated among the 23 children who received the standard GCV dose of 5 mg/kg bid was 38.3 mg/L·h (24.8–329.2). More than half (13/23) of the children had an AUC_ss,24h_ <40 mg/L·h (Table S4). The simulations showed that 61% of children (*n* = 703) would achieve the target AUC_ss,24h_ with standard GCV dosing: young children aged 1–4 years with normal SCR values of 10–50 μmol/L having low drug exposure (25.8 mg/L·h (18.2–46.2)) (Fig. 4).

### Dose regimen recommendation

Based on the Monte Carlo simulation, an optimized dosing regimen for GCV was determined stratified by renal function and age (Table 4). This new dosing regimen for children with normal renal function had a PTA of achieving an AUC_ss,24h_ of 40–100 mg/L·h between 61% and 78% across subgroups. The standard GCV dose was associated with a PTA of 50% (Table S6), with the optimized dosing regimen improving this to 70%.

**TABLE 4:** Optimized ganciclovir dosing regimen for children with normal serum creatinine^*a*^

Age	Serum creatinine (μmol/L)	Optimized dosing regimen	PTA (%)	
			Optimized dosing	Standard dosing
1–4 years	10–30	13 mg/kg bid	61.0	7.8
	30–50	7 mg/kg bid	75.3	51.7
4–10 years	20–40	7 mg/kg bid	67.5	43.7
	40–60	5 mg/kg bid	75.7	77.1
10–16 years	30–60	5 mg/kg bid	74.3	72.4
	60–80	3 mg/kg bid	76.3	47.2
16–18 years	50–80	3 mg/kg bid	78.1	45.3
	80–110	2 mg/kg bid	77.7	13.0

^*a*^ PTA: probability of target attainment. The age-related normal serum creatinine distribution was derived from a covariate database of The Royal Children’s Hospital Melbourne.

## DISCUSSION

In our study, only 61% of children achieved a serum GCV AUC_ss,24h_ of 40–100 mg/L·h with standard IV GCV dosing with young children, aged 1–4 years, having the lowest PTA. Given the high morbidity and mortality associated with CMV infection, these data support the need for: (i) routine therapeutic drug monitoring; and (ii) revision of the current pediatric GCV dosing strategy. Our optimized dosing regimen had a PTA of 61%–78% in achieving an AUC_ss,24h_ of 40–100 mg/L·h in children with normal renal function and should be evaluated in a clinical cohort. This new regimen recommends higher than standard doses for young children aged <4 years and lower doses for older children aged >16 years (Fig. 4; Table S5). A potential reason for the lower doses required in children aged >16 years could be that the majority of our population were SCT recipients. Selby et al. similarly found that adult SCT recipients also required lower doses (3.75 mg/kg qd) (27). This was also observed in our review of published GCV PopPK models that showed that the standard 5 mg/kg bid dose could potentially lead to overexposure (AUC_24_ >120 mg/L·h) in three of six studies (15).

PK/PD studies of GCV for CMV infection remain scarce, especially in children (15, 28, 29). Although previous PK studies of GCV have targeted an AUC_ss,24h_ of 80–120 mg/L·h for CMV treatment, this target is based on expert opinion alone, with no original data correlating this therapeutic range with improved clinical outcomes of CMV infection (26–28). Of concern, a study in adults reported a higher probability of GCV-related leukopenia with this therapeutic target (30% for AUC_ss,24h_ 80–120 mg/L·h vs 10% AUC_ss,24h_ 40–60 mg/L·h), a risk that may potentially outweigh a benefit in efficacy (30, 31). For CMV prophylaxis, many PopPK studies have targeted an AUC_ss,24h_ of 40–50 or 40–60 mg/L·h (15). These targets have primarily been extrapolated from adult data (9). The usual approach to defining a therapeutic target for children is to aim for a similar drug exposure to that observed in adults (32). For GCV, an IV dose of 5 mg/kg bid in adults typically achieves a median AUC_ss,24h_ range of between 50 and 90 mg/L·h, although a wide variability has been reported (28, 33, 34). Breakthrough CMV infection in patients receiving GCV prophylaxis has been reported with an AUC_ss,24h_ below 40 mg/L·h (9, 10), so targeting a drug exposure above this threshold is prudent. Based on this evidence, a GCV AUC_ss,24h_ of 40–100 mg/L·h was adopted as the therapeutic target in our study.

Several published PopPK studies have proposed alternative dosing regimens in children. Li et al. (25) recommended a GCV dosing regimen based on estimated glomerular filtration rate (eGFR) and weight subgroups. However, this study used a therapeutic target of AUC_ss,24h_ > 40 mg/L·h without considering an upper limit: this may lead to an increased risk of toxicity. A weight-based dosing regimen based on CrCL and age was recommended by Franck et al. (8) targeting an AUC_ss,24h_ of 40–60 mg/L·h. The recommended dosing range was 5–10 mg/kg bid for children with normal CrCL, whereas that of the optimized dosing regimen in our study was 2–13 mg/kg bid for children with normal SCR to target an AUC_ss,24h_ of 40–100 mg/L·h. Nguyen et al. (26) proposed a daily IV GCV dose of 15 to 20 mg/kg/day for children with normal eGFR and 20 to 25 mg/kg/day GCV in those with augmented eGFR. Notably, the therapeutic target in this study was higher (AUC_0-24h_ 80–120 mg/L·h). All of these studies highlight the need to improve the GCV dosing strategy for CMV infection in children.

The transferability of five published PopPK models was limited for our population of immunocompromised children. The model by Zhou et al. (23) overpredicted our data: using this model, a typical patient in our study weighing 27.4 kg with an SCR of 30 µmol/L (CrCL = 153 mL/min/1.73 m^2^) would have a CL of 0.68 L/h. This value is unusually low for a child (25, 35), leading to high simulated concentrations evident in the visual predictive check (VPC) plot (Fig. S3). Body size is an important covariate for GCV PK in children; however, body weight was not included on CL in this model, which might result in poor transferability. Although renal function significantly influences GCV elimination, Acosta et al. (24) did not identify an effect of renal function on CL, and their model was mainly for neonates aged less than 1 month, which could explain the poor transferability to our patient cohort. Due to large differences in population characteristics (median age, 2.46 years [range, 0.1–12.8]; median weight 12.0 kg [range, 2.5–55.0]; and critically ill patients), the model by Li et al. (25) could not describe our data well. The external validation results of Franck et al. (8) and Nguyen et al. (26) demonstrated relatively better alignment, likely due to certain shared characteristics among study populations. The high PK variability of GCV observed across different pediatric populations underscores the need for ongoing research in this area.

Because GCV is renally cleared, it is expected that renal replacement therapy (RRT) influences GCV pharmacokinetics. Hemodialysis has been shown to significantly reduce GCV concentrations by approximately 50% (36). In our study, four children received RRT during their treatment episode: these children were not excluded in the external validation and model building (Table S1) as three of these children contributed data while both receiving and not receiving RRT. RRT was considered as a covariate for CL during model development; however, the OFV decreased by only 0.056, and the CL during RRT was 0.981 of the CL of those who were not receiving RRT. Plots of individual predictions versus observations stratified by whether the patient received RRT showed no intra-individual variability attributable to RRT (Fig. S4). Potential reasons for this were the limited number of samples available for those receiving RRT (24/161), one patient lacked pre-and post-RRT values and the relatively short duration of RRT within the total treatment course for the remaining three patients (Fig. S5).

A limitation of our study is the relatively small number of included children, which may affect the generalizability of study results. Another limitation was the use of retrospective therapeutic drug monitoring data for model development; however, each child had two timed blood samples taken to determine the AUC_24_, and clinical staff were trained on the accurate documentation of the timing of dosing and blood sampling. Although previous studies have used two-compartment models to describe GCV PopPK, a one-compartment model was selected in this study due to the relatively limited sampling times over the dosing interval. This may have led to inaccuracy in the estimation of AUC_24_. Although SCR could be affected by different factors, including age, muscle mass, and diet, this study used SCR as a measure of renal function as it reflects clinical practice (37). Also, recent measurements of height were not available for the majority of patients, limiting our ability to accurately estimate CrCL. It should also be noted that the two children in this study contributed more data than the rest of the cohort. A sensitivity analysis showed that the parameter estimates were comparable with and without these two children included (Table S7). Further analysis confirmed that the final dose recommendations remained similar and retaining these data enhanced the reliability of parameter estimation (Table S8). Finally, in the absence of high-quality evidence, we used a therapeutic target of AUC_ss,24h_ 40–100 mg/L·h, a PK/PD target that requires further investigation.

### Conclusions

Our study suggests that standard IV GCV dosing of 5 mg/kg twice daily results in inadequate drug exposure in approximately half of the children, particularly those aged <4 years. This study adds to the growing body of evidence highlighting the need for routine GCV therapeutic drug monitoring for children and to evaluate new empirical dosing strategies to reduce mortality, morbidity, and treatment-related toxicity associated with CMV infection in immunocompromised children. Our optimized dosing regimen had a PTA of achieving an AUC_ss,24h_ of 40–100 mg/L·h of 61%–78% and should be evaluated in future prospective clinical studies. Further clinical trials with optimal pharmacokinetic sampling strategies and minimally invasive sampling methods (e.g., micro-sampling) are needed to further characterize the pharmacokinetics of VGCV/GCV, in particular in those children receiving renal replacement therapy.

## Data Availability

The data generated during and/or analyzed during the current study are available from Adam Irwin or Amanda Gwee on reasonable request.

## SUPPLEMENTAL MATERIAL

Supplemental material aac.00525-24-s0001.docx Tables S1 to S8, Figures S1 to S5, and supplemental methods.

ASM does not own the copyrights to Supplemental Material that may be linked to, or accessed through, an article. The authors have granted ASM a non-exclusive, world-wide license to publish the Supplemental Material files. Please contact the corresponding author directly for reuse.
