# Supplementary material for: Serum ganciclovir drug exposure in children receiving standard ganciclovir dosing
Source: Antimicrob Agents Chemother. 2024 Sep 18;68(10):e00525-24. doi: 10.1128/aac.00525-24 (PMC11459965; doi:10.1128/aac.00525-24)
Supplement: Supplemental material — Tables S1 to S8, Figures S1 to S5, and supplemental methods. [file aac.00525-24-s0001.docx]

**Serum ganciclovir drug exposure in children receiving standard ganciclovir dosing**

**Authors:**

Wenyu Yang ^a^, Adam Irwin ^b, c^, Heather Weerdenburg ^d, e, f^, Brett McWhinney ^g^, Theresa Cole ^h^, Alice Lei ^i^, Bing Han ^j,^ *, Xiao Zhu ^a,^ *, Amanda Gwee ^e, f, i^.

**Affiliations:**

^a^ Minhang Hospital & School of Pharmacy, Fudan University, Shanghai, China.

^b^ Centre for Clinical Research, The University of Queensland, Brisbane, Queensland, Australia.

^c^ Infection Management and Prevention Service, Queensland Children’s Hospital, Brisbane, Queensland, Australia.

^d^ Children's Cancer Centre, Royal Children's Hospital, Melbourne, Victoria, Australia

^e^ Department of Paediatrics, The University of Melbourne, Melbourne, Victoria, Australia.

^f^ Antimicrobial Group, Murdoch Children's Research Institute, Victoria, Australia.

^g^ Department of Chemical Pathology, Pathology Queensland, Royal Brisbane and Women’s Hospital, Brisbane, Queensland, Australia.

^h^ Department of Haematology, Royal Children's Hospital, Melbourne, Victoria, Melbourne, Australia

^i^ Department of General Medicine, Royal Children's Hospital, Melbourne, Victoria, Australia.

^j^ Department of Pharmacy, Minhang Hospital, Fudan University, Shanghai, China.

**Emails:**

Wenyu Yang: [21211030109@m.fudan.edu.cn](mailto:21211030109@m.fudan.edu.cn)

Adam Irwin: [a.irwin@uq.edu.au](mailto:a.irwin@uq.edu.au)

Heather Weerdenburg: [Heather.Weerdenburg@rch.org.au](mailto:Heather.Weerdenburg@rch.org.au)

Brett McWhinney: [Brett.McWhinney@health.qld.gov.au](mailto:Brett.McWhinney@health.qld.gov.au)

Theresa Cole: [Theresa.Cole@rch.org.au](mailto:Theresa.Cole@rch.org.au)

Alice Lei: [Alice.Lei2@rch.org.au](mailto:Alice.Lei2@rch.org.au)

Bing Han: [hbshcn@fudan.edu.cn](mailto:hbshcn@fudan.edu.cn)

Xiao Zhu: [xiaozhu@fudan.edu.cn](mailto:xiaozhu@fudan.edu.cn)

Amanda Gwee: [Amanda.Gwee@rch.org.au](mailto:Amanda.Gwee@rch.org.au)

***Corresponding authors:**

Xiao Zhu, Minhang Hospital & School of Pharmacy, Fudan University, 201203, Shanghai, China. Tel: +86 21 51980024. Email: [xiaozhu@fudan.edu.cn](mailto:xiaozhu@fudan.edu.cn)

Bing Han, Department of Pharmacy, Minhang Hospital, Fudan University, No.170 Xinsong Road, 201199, Shanghai, China. Tel: +86 189 1816 9079. Email: [hbshcn@fudan.edu.cn](mailto:hbshcn@fudan.edu.cn)

Contents

[Patient data: result 3](#_Toc175694660)

[External validation of published pediatric models: methods and results 3](#_Toc175694661)

[Population pharmacokinetic model: methods and results 8](#_Toc175694662)

[Drug exposure evaluation: results 11](#_Toc175694663)

[Dose optimization: results 12](#_Toc175694664)

[Analysis of renal replacement therapy: results 12](#_Toc175694665)

[Sensitivity Analysis after removing 2 patients’ data: results 14](#_Toc175694666)

# Patient data: result

**Table S1.** Biochemical characteristics of the population at baseline and chronology of 4 children received RRT.

| **Characteristics** | **Number of patients (%)**  **Median [min-max]** | |
| --- | --- | --- |
| White cell count (×10^9/L) | 4.1 [0.1 – 13.9] * | |
| Neutrophils (×10^9/L) | 2.06 [0.05 – 11.9] * | |
| Alanine transaminase (U/L) | 47.0 [15.0 – 1700] * | |
| Albumin (g/L) | 28.0 [16.0 – 38.0] * | |
| **The chronology of 4 children received RRT** | **Number of samples in RRT/ total** | **Duration of RRT/ total duration (days)** |
| ID2 | 2/2 | 34.9/34.9 |
| ID33 | 3/5 | 32.1/52.6 |
| ID3369 | 9/53 | 20.0/129.4 |
| ID5012 | 10/44 | 13.6/99.7 |
| *: 3 patients without this covariate information.  RRT: renal replacement therapy. | | |

# External validation of published pediatric models: methods and results

**Methods**

Published pediatric GCV/VGCV pharmacokinetic models were selected according to the following diagram.


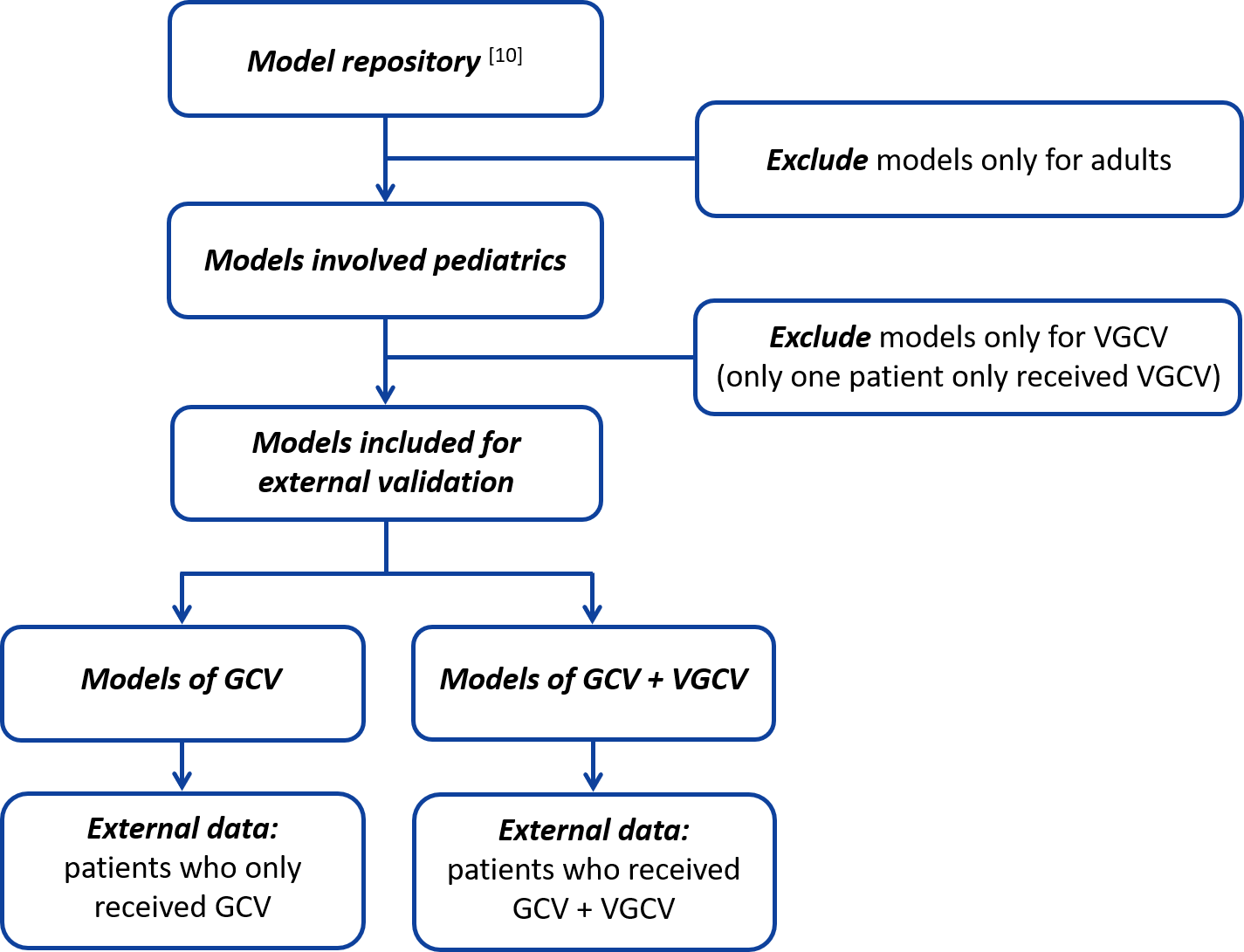


**Figure S1.** Workflow of model screening for external validation. GCV: ganciclovir; VGCV: valganciclovir.

To evaluate the published models, goodness-of-fit (GOF) plots were first assessed, and mean prediction error (MPE) and mean absolute prediction error (MAPE) were calculated to assess the bias between population predictions (PRED) and observations. Additionally, root mean square error (RMSE) was also calculated to assess the precision of the models. Visual predictive check (VPC, n=1000) was visualized with the R package "tidyvpc".

$$MPE=\frac{1}{N}\times\sum_{i=1}^{N} ({pred}_{i}-{obs}_{i})$$

$$MAPE=\frac{1}{N}\times\sum_{i=1}^{N} (|{pred}_{i}-{obs}_{i}|)$$

$$RMSE=\sqrt{\frac{1}{N}\times\sum_{i=1}^{N} {({pred}_{i}-{obs}_{i})}^{2}}$$

where N stands for the number of observations and *obs_i_* denotes the *i*^th^ observation.

**Results**

**Table S2.** Characteristics of models included in external validation

| **Study** | **Subjects (observations)** | **Population** | **Drug** | **Age Median [Range]** | **Weight (kg) Median [Range]** | **Renal function**  **Median [Range]** | **Model structure** | **Fixed effect parameters** | |
| --- | --- | --- | --- | --- | --- | --- | --- | --- | --- |
| Nguyen et al.[25] | 105 (374) | Pediatrics | GCV: i.v.  VGCV: p.o. | 2.5 years  [0.01 - 17.3] | 11.7  [2.6 - 80] | eGFR (mL/min/1.73m^2^):  167 [43 - 425] | 2-comp | CL (L/h)=2.55×(BW/11.7)^0.75^×(eGFR/167)^0.763^×0.806^critically ill^  Vc (L)=5.96×(BW/11.7)  Q (L/h)=0.222×(BW/11.7)^0.75^  Vp (L)=1.29×(BW/11.7)  Ka (1/h)=0.506  *F=0.438 (absolute bioavailability = 0.608) | |
| Franck et al.[8] | 50 (580) | Pediatrics received SOT and SCT | GCV: i.v.  VGCV: gastrostomy and nasogastric tube | 7.5 years  [0.5 - 17.4] | 26.7  [5.96 - 87] | CrCL (mL/min/1.73m^2^):  150 [56 - 345] | 2-comp | CL (L/h)=6.9×(BW/26.7)^0.75^×(CrCL/149.8)^0.88^  Vc (L)=9.7×(BW/26.7)  Q (L/h)=10.9  Vp (L)=7.6×(BW/26.7)  Tlag (h)=0.33  Ka (1/h)=0.73  *F=0.43 (absolute bioavailability = 0.597) | |
| Li et al.[24] | 104 (138) | Critically ill pediatrics | GCV: i.v. | 2.46 years  [0.1 - 12.8] | 12.0  [2.5 - 55.0] | eGFR (mL/min/1.73m^2^):  111 [15 - 129]  KF:  0.92 [0.12 - 1.08] | 1-comp | CL (L/h)=5.23×KF^0.92^×(BW/12.0)^1.02^  V (L)=11.35×(BW/12.0)^0.80^ | |
| Acosta et al.[23] | 24 (484) | Neonates with symptomatic congenital CMV disease | GCV: i.v.  VGCV: p.o. | study1.0:  30 days  [11 - 34]  study2.0:  20 days  [8 - 33] | study1.0:  2.7  [2.1 - 3.4]  study2.0:  2.9  [1.9 - 4.4] | NR | 1-comp | CL (L/h)=0.146×BW^1.68^  V (L)=1.15×BW  Ka (1/h)=0.591  *F=0.536 (absolute bioavailability = 0.744) | |
| Zhou et al.[22] | 27 (219) | Newborns with acute symptomatic CMV disease | GCV: i.v. | NR | NR | ASCC (mL/min/1.73m^2^):  NR | 1-comp | CL (L/h)=0.262+(0.00271×ASCC)  V (L)=0.627+(0.437×BW) | |
| *: these estimates of F had not been adjusted by 0.72 (the ratio between the molecular weights of GCV and VGCV), so the absolute bioavailability was calculated by F/0.72.  ASCC: approximated serum creatinine clearance, calculated by modified Schwartz formula; BW: body weight; CL: clearance; CMV: cytomegalovirus; CrCL: creatinine clearance, calculated by modified Schwartz formula; eGFR: estimated glomerular filtration rate, calculated by modified Schwartz formula; F: bioavailability; GCV: ganciclovir; i.v.: intravenous infusion administration; Ka: absorption rate constant; KF: kidney function, a dimensionless parameter, calculated as dividing eGFR by the normal renal function (120 mL/min/1.73 m^2^); p.o.: oral administration; Q: intercompartment clearance; SCT: stem cell transplant; SOT: solid organ transplant; Tlag: lag time; Vc: volume of distribution of central compartment; VGCV: valganciclovir; Vp: volume of distribution of peripheral compartment. | | | | | | | | |  |


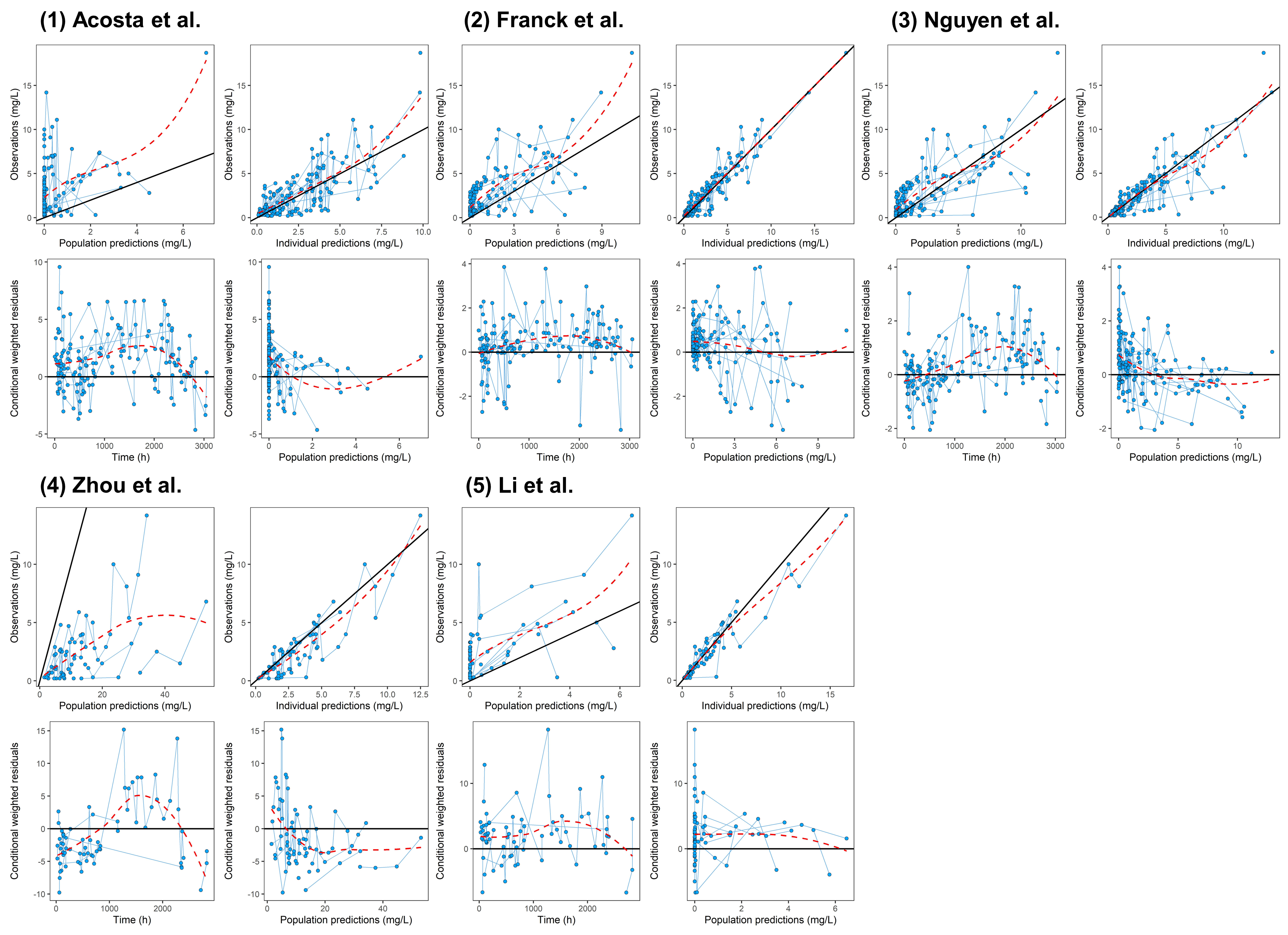


**Figure S2.** Goodness-of-fit plot of published models. Row 1: three models for IV GCV or oral VGCV; Row 2: two models for IV GCV.


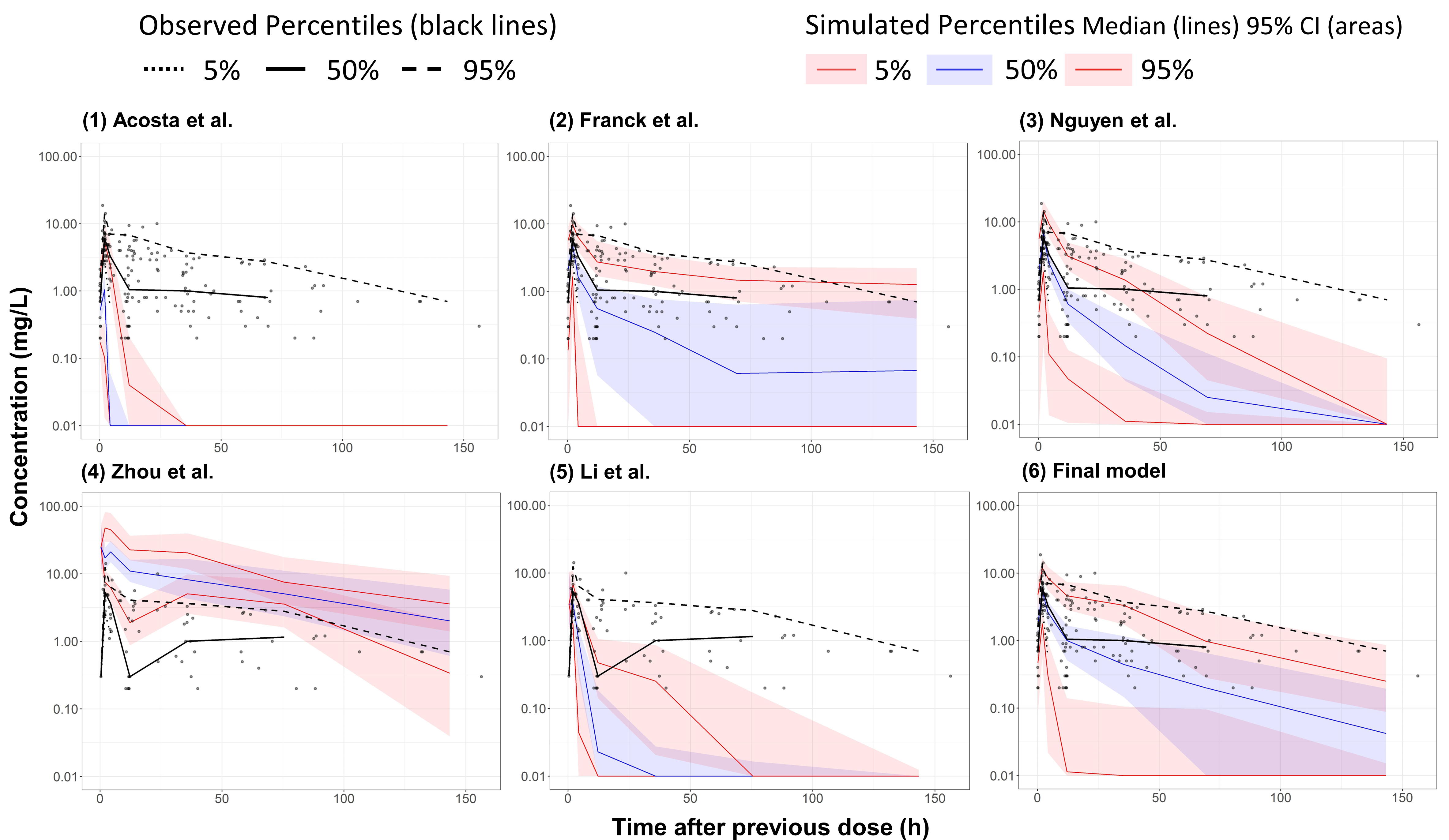


**Figure S3.** Visual predictive check (VPC) plots of included models. Row 1: three models for IV GCV or oral VGCV; Row 2: two models for IV GCV. The black line represents the median observed data and the two black dashed lines represent the 5th and the 95th percentages of observed data, respectively. The light blue and light red areas represent the 95% confidence interval for the median and the 5th and 95th percentiles of the simulated data, respectively.

# Population pharmacokinetic model: methods and results

**Methods**

The final model was evaluated using GOF plots and VPC. The final model was assessed using bootstrap analysis (n=1000) and the median estimates along with 95% confidence intervals determined.

Exploration of covariate effect on exposure: R package "coveffectsplot" (version 1.0.2, <https://github.com/smouksassi/coveffectsplot>). This analysis involved creating a template dataset in which one covariate was systematically altered at a time, while keeping all other covariates consistent with their reference values. For continuous covariates, the reference value was set to the median of the covariate, and the effects were assessed at specific covariate values, including the 5^th^, 25^th^, 75^th^, and 95^th^ percentiles. Categorical covariates were examined by considering all categories, with one category designated as the reference. The template dataset included all possible combinations of covariate values. Using the standard dose regimens of GCV (5 mg/kg twice daily, infused over 1 hour), 1000 PK profiles were simulated for each unique covariate combination. The fixed effect parameters were sampled from the uncertainty distribution derived from the final model's variance-covariance matrix. AUC_ss,24h_ was then computed on each profile for each subject.

**Results**

The IIV was optimally described by an exponential model. However, the data did not provide sufficient information to estimate IIV for volume of distribution of the central compartment (V), F, and absorption rate constant (Ka). Fixing the variance of these random effects to zero did not impact the OFV. Residual unexplained variability (RUV) could be best expressed by a combined error model. Though the structural models of both one- and two-compartment displayed comparable GOF plots, the one-compartment model had a smaller AIC (644.151 vs. 645.884). Along with the principle of parsimony, the one-compartment model was chosen.

Given the significant influence of renal function on GCV's CL, we first tested the effects of renal function. Adding the effect of SCR on CL decreased the OFV by 153.22 points. Considering both the findings from published models and the ease of using weight in clinical practice, weight was included in the SCM process. In the forward inclusion process, only weight on CL and V produced a significant decrease in the OFV. After the backward exclusion process, they were retained in the final model. In the final model, fixed allometric scaling factors were applied, with both CL and V scaled to a reference individual weighing 70 kg. This approach led to a reduction of 0.378 in the AIC, ensuring the model maintained physiological relevance and enhancing its comparability across different models

**Table S3. Development of the final population pharmacokinetic model.**

|  | **Structural model** | | **Base model** | **Covariate model** | | | | **Final model** |
| --- | --- | --- | --- | --- | --- | --- | --- | --- |
|  | **1-comp** | **2-comp** | **1-comp + SCR on CL** | **1-comp + SCR on CL + WT on CL** | **1-comp + SCR on CL + WT on CL+ WT on V1** | **1-comp + SCR on CL + WT on CL+ WT on V1 + fix IIV-V1 to 0** | **1-comp + SCR on CL + WT on CL+ WT on V1 + fix IIV-V1 to 0 + fix F to 0.536** | **1-comp + SCR on CL + fixed allometric scaling factors + fix IIV-V1 to 0 + fix F to 0.536** |
| AIC | 644.151 | 645.884 | 492.931 | 467.062 | 426.795 | 424.793 | 427.492 | 427.114 |
| OFV | 332.253 | 329.986 | 179.033 | 151.164 | 108.897 | 108.895 | 113.594 | 117.216 |
| Minimization ^a^ | S, C | S, B | S, C | S, C | S, B | S, C | S, C | S. C |
| **Fixed effect parameters: Estimates (RSE)** | | | | | | |  |  |
| Ka (h^-1^) | 0.926 (34%) | 0.854 | 0.624 (19%) | 0.637 (16%) | 0.577 | 0.577 (14%) | 0.641 (28%) | 0.660 (25%) |
| F ^b^ | 0.605 (114%) | 0.594 | 0.700 (56%) | 0.694 (67%) | 0.675 | 0.674 (46%) | 0.536 FIX | 0.536 FIX |
| CL (L/h) | 4.7 (19%) | 4.63 | 6.79 (19%) | 7.15 (10%) | 7.37 | 7.37 (8%) | 7.1 (9%) | 14.1 (9%) |
| V1 (L) | 26.8 (34%) | 25.8 | 23.0 (24%) | 23.2 (22%) | 25.6 | 25.6 (7%) | 24.4 (7%) | 62.5 (4%) |
| Q (L/h) | / | 0.1 | / | / | / | / | / | / |
| V2 (L) | / | 11.6 | / | / | / | / | / | / |
| SCR on CL | / | / | 0.981 (9%) | 1.03 (7%) | 1.02 | 1.02 (7%) | 1.02 (5%) | 0.997 (7%) |
| WT on CL | / | / | / | 0.946 (15%) | 0.984 | 0.984 (15%) | 0.987 (12%) | 0.75 FIX |
| WT on V | / | / | / | / | 1.05 | 1.05 (6%) | 1.03 (9%) | 1 FIX |
| **Inter-individual variability (CV%): Estimates (RSE) [Shrinkage]** | | | | | | |  |  |
| CL | 73.6% (11%) [17%] | 74.0% [17%] | 74.2% (21%) [8%] | 33.6% (18%) [22%] | 33.0% [12%] | 33.0% (14%) [12%] | 33.3% (18%) [12%] | 36.6% (15%) [10%] |
| V1 | 72.2% (20%) [34%] | 73.9% [33%] | 72.2% (21%) [21%] | 68.0% (20%) [18%] | 0.3% [99%] | / | / | / |
| **Residual unexplained error: Estimates (RSE) [Shrinkage]** | | | | | | |  |  |
| Prop.error | 0.507 (14%) [7%] | 0.513 [7%] | 0.396 (8%) [9%] | 0.393 (8%) [8%] | 0.382 [5%] | 0.382 (7%) [5%] | 0.391 (7%) [5%] | 0.392 (7%) [5%] |
| Add.error (mg/L) | 0.742 (35%) [7%] | 0.701 [7%] | 0.165 (22%) [9%] | 0.169 (22%) [8%] | 0.160 [5%] | 0.160 (19%) [5%] | 0.147 (25%) [5%] | 0.144 (21%) [5%] |
| a: S=Minimization successful; B=Parameter estimates near boundary; C=Covariance step successful.  b: F, bioavailability, included the ratio between the molecular weights of GCV and VGCV (which equals to 0.72).  CL: clearance; CV%: coefficient of variability, expressed as $\sqrt{{omega}^{2}}\times100\%$; IIV: inter-individual variability; Ka: absorption rate constant; Q: Intercompartment clearance from central compartment to first peripheral compartment; RSE: relative standard error; SCR: serum creatinine (μmol/L); V1: central compartment volume of distribution; V2: peripheral compartment volume of distribution; WT: body weight. | | | | | | | | |

# Drug exposure evaluation: results

**Results**

**Table S4.** Individual ganciclovir exposure of 23 children under standard ganciclovir dosing.

| **No.** | **Age** | **Weight** | **Serum creatinine** | **Drug** | **Dose (mg)** | **AUC_24h_** |
| --- | --- | --- | --- | --- | --- | --- |
| 1 | 4 | 16 | 26 | GCV | 80 | 30.9 |
| 2 | 4 | 16.2 | 30 | GCV | 81 | 39.1 |
| 3 | 0.4216 | 6.9 | 22 | GCV | 34 | 17.3 |
| 4 | 17 | 69.4 | 73 | GCV | 347 | 63.3 |
| 5 | 13 | 73.3 | 150 | GCV | 366 | 346.6 |
| 6 | 11 | 31.7 | 30 | GCV | 158 | 36.5 |
| 7 | 9 | 25.4 | 60 | GCV | 127 | 52.7 |
| 8 | 9 | 35.7 | 28 | GCV | 178 | 33.9 |
| 9 | 9 | 30.9 | 93 | GCV | 154 | 95.8 |
| 10 | 10 | 28.2 | 39 | GCV | 141 | 48.8 |
| 11 | 9 | 32.3 | 32 | GCV | 162 | 33.9 |
| 12 | 5 | 26.5 | 149 | GCV | 132 | 172.2 |
| 13 | 3 | 13.7 | 20 | GCV | 68 | 26.7 |
| 14 | 14 | 50.6 | 21 | GCV | 253 | 31.8 |
| 15 | 2 | 13.2 | 22 | GCV | 66 | 34.3 |
| 16 | 9 | 28.6 | 19 | GCV | 143 | 24.6 |
| 17 | 0.42 | 5.61 | 23 | GCV | 28 | 38.3 |
| 18 | 9 | 19.94 | 34 | GCV | 100 | 40.4 |
| 19 | 10 | 37.7 | 83 | GCV | 188 | 169.5 |
| 20 | 2 | 12 | 30 | GCV | 60 | 31.6 |
| 21 | 16 | 67.8 | 167 | GCV | 339 | 471 |
| 21 | 2 | 11.95 | 30 | GCV | 60 | 37.2 |
| 23 | 14 | 67.8 | 97 | GCV | 339 | 73.9 |

**Table S5.** Simulated ganciclovir exposure (n=703) for different age groups with normal renal function under standard ganciclovir dosing.

| **Age group** | **Median [5^th^-95^th^ percentiles] AUC_ss,24h_** | **% achieved AUC_ss,24h_ of 40-100 mg/L** |
| --- | --- | --- |
| 1-4 years  10-50 μmol/L | 25.8 [18.2-46.2] | 8.0 |
| 4-10 years  20-60 μmol/L | 44.5 [28.1-69.2] | 63.7 |
| 10-16 years  30-80 μmol/L | 65.3 [46.4-107.6] | 91.6 |
| 16-18 years  50-110 μmol/L | 99.65 [78.1-145.3] | 50.0 |
| Total | 51.4 [22.2-109.7] | 60.6 |

# Dose optimization: results

**Table S6.** Percentage of pediatrics with normal renal function achieving an AUC_ss,24h_ of 40-100 mg/L·h under standard and optimized ganciclovir dosing

| **Dosing approach** | **Group** | **PTA (%)** | **AUC_ss,24h_ (mg/L·h)**  **Median [5^th^-95^th^ percentiles]** |
| --- | --- | --- | --- |
| **^a^ Standard dose regimen** | **Total** | **49.8** | **50.3 [17.5-141.9]** |
|  | 1-4 years old | 22.4 | 27.6 [13.4-60.3] |
|  | 4-10 years old | 53.3 | 43.7 [20.7-93.5] |
|  | 10-16 years old | 67.3 | 68.3 [31.6-140.6] |
|  | 16-18 years old | 46.4 | 102.7 [52-205.6] |
| **Initial dose recommendation** | **Total** | **69.6** | **59.2 [29.2-121.1]** |
|  | 1-4 years old | 70.9 | 60.8 [30.2-122.6] |
|  | 4-10 years old | 69.0 | 55.7 [27.3-110] |
|  | 10-16 years old | 72.4 | 62.5 [30.9-127.7] |
|  | 16-18 years old | 73.8 | 59.1 [30.5-111.4] |
| ^a^: The standard dosing regimen for ganciclovir is 5 mg/kg twice daily with an infusion over 1 hour. PTA: probability of target attainment. | | | |

# Analysis of renal replacement therapy: results


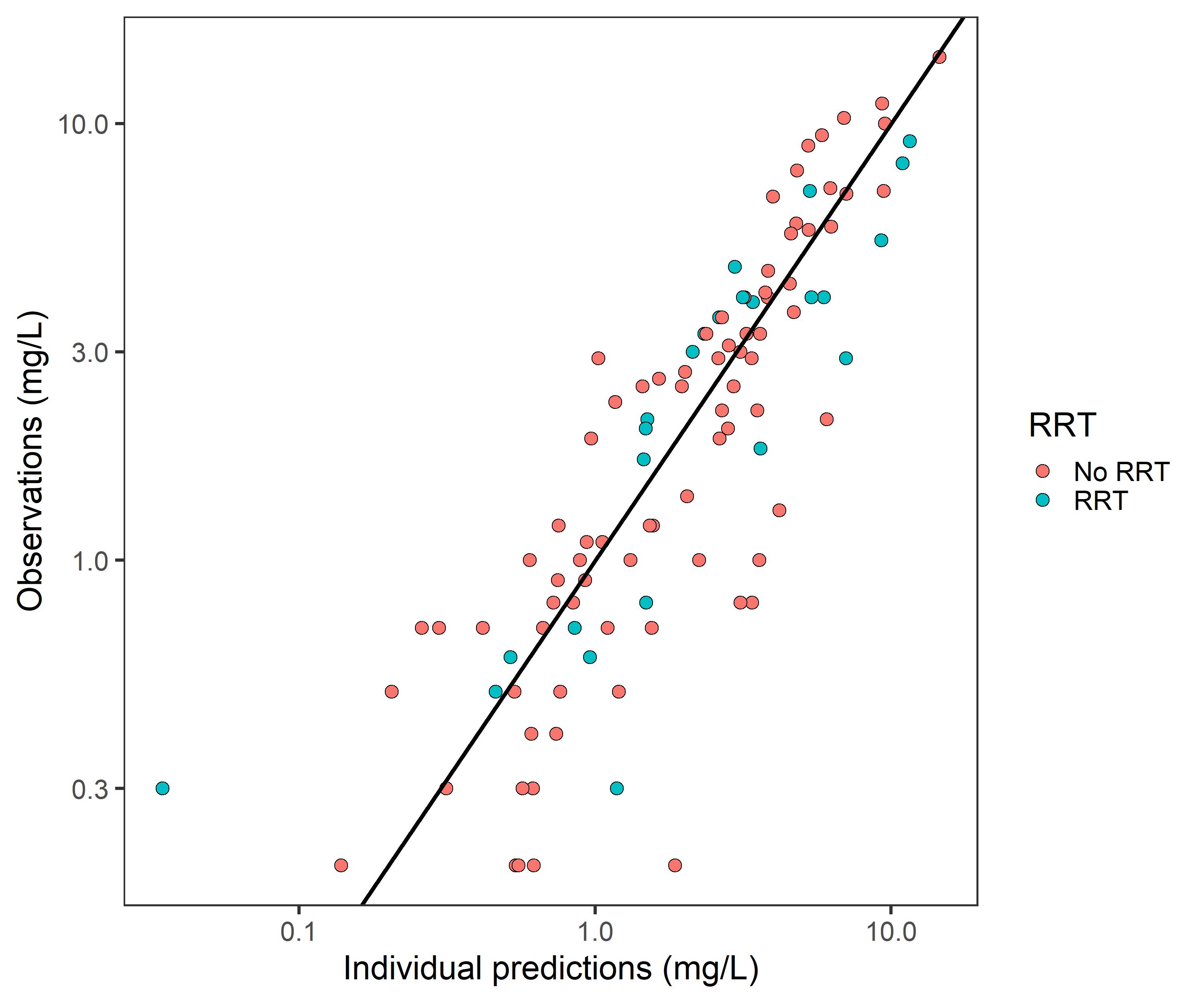


**Figure S4. The scatter plot of individual predictions versus observations for 4 children receiving renal replacement therapy (RRT).**





**Figure S5. The time courses for 4 children receiving renal replacement therapy (RRT).** Orange points represent observations of ganciclovir level. Black lines represent the periods without RRT, blue lines represent the periods receiving continuous RRT (CRRT), and red lines represent the periods receiving hemodialysis.

# Sensitivity Analysis after removing 2 patients’ data: results

**Table S7.** Parameter estimation difference statistical results (Wald test).

| **Parameter** | **Estimate (RSE)** | | **p value** |
| --- | --- | --- | --- |
|  | **Before** | **After** |  |
| CL (L/h) | 14.1 (9%) | 13.5 (10%) | 0.748 |
| The effect of SCR on CL | 0.997 (7%) | 0.739 (13%) | 0.0324 |
| V (L) | 62.5 (4%) | 65.6 (8%) | 0.5942 |
| Ka (h^-1^) | 0.66 (25%) | 0.78 (44%) | 0.752 |

**Table S8.** Comparison of the final dose recommendations.

| **Age** | **Serum creatinine (****μmol/L)** | **Optimized dose (mg/kg bid)** | | **PTA (%)** | |
| --- | --- | --- | --- | --- | --- |
|  |  | **before** | **after** | **before** | **after** |
| 1 - 4 years | 10-30 | 13 | 13 | 61.0 | 74.1 |
|  | 30-50 | 7 | 7 | 75.3 | 77.8 |
| 4 - 10 years | 20-40 | 7 | 7 | 67.5 | 74.3 |
|  | 40-60 | 5 | 5 | 75.7 | 77.1 |
| 10 - 16 years | 30-60 | 5 | 5 | 74.3 | 79.5 |
|  | 60-80 | 3 | 4 | 76.3 | 79.0 |
| 16 - 18 years | 50-80 | 3 | 3 | 78.1 | 77.5 |
|  | 80-110 | 2 | 3 | 77.7 | 78.9 |
